# Effectiveness of Story-Centred Care Intervention Program in older persons living in long-term care facilities: A randomized, longitudinal study

**DOI:** 10.1371/journal.pone.0194178

**Published:** 2018-03-19

**Authors:** Hui-Wan Chuang, Chi-Wen Kao, Ming-Der Lee, Yue-Cune Chang

**Affiliations:** 1 Graduate Institute of Medical Sciences, National Defense Medical Center, Taipei, Taiwan; 2 Department of Nursing, Tri-Service General Hospital, Taipei, Taiwan; 3 School of Nursing, National Defense Medical Center, Taipei, Taiwan; 4 Graduate Institute of Long-Term Care, National Taipei University of Nursing and Health Sciences, Taipei, Taiwan; 5 Department of Mathematics, Tamkang University, New Taipei City, Taiwan; Brown University, UNITED STATES

## Abstract

Depression is a common issue in institutionalized elderly people. The “Attentively Embracing Story” theory is applied to help individuals transform negative thoughts into positive, and reflect on spiritual healing. This study aimed to examine the effectiveness of a “Story-Centred Care Intervention Program” based on the “Attentively Embracing Story” theory in improving depressive symptoms, cognitive function, and heart rate variability in institutionalized elderly people. Seventy long-term care residents were recruited from two long-term care facilities and randomized into the story-centred care intervention (n = 35) and control groups (n = 35). We excluded five long-term care residents who did not complete the post-test measures and five long-term care residents who had interference events on the outcome measures. Finally, sixty long-term care residents (40 women and 20 men; age 84.3±5.98 years) were included in the final analysis. Data were collected at four times (pre-intervention and post-intervention, 1 and 3-month follow-up) and analyzed with the generalized estimating equation approach.Instruments, including Geriatric Depression Scale, Short Portable Mind Status Questionnaire, and a CheckMyHeart device to measure heart rate variability, were used in study. The degree of improvement in depressive symptoms was significantly higher in the story-centred care intervention group than in the control group after providing the story-centred care intervention program (p < .001) and at 1 and 3-month follow-up (p = .001, p = .006, respectively; GDS-15 score reduced 1.816 at the 3-month follow-up). Participants receiving the story-centred care intervention program showed significantly greater improvement than those in the control group in the cognitive function at 1and 3-month follow-up (p = .009, p = .024, respectively; SPMSQ score reduced 0.345 at the 3-month follow-up). The heart rate variability parameters (SDNN, RMSSD) did not show a statistically significant increase. However an increasing trend in the parameters was observed in the intervention group (SDNN increased 16.235ms at the 3-month follow-up; RMSSD increased 16.424 ms at the 3-month follow-up). In conclusions, the story-centred care intervention program was effective on the improvement of depressive symptoms and cognitive status in institutionalized elderly people.

## Introduction

Aging adults experience increased chronic diseases, and reduced physical activity, which requires more assistance and care from others [1, 2]. In Taiwan, families are the main caregivers for older adults [3]. Older adults cared for in institutional settings have fewer social interactions and support from family and friends [4], which could influence physical and mental health [5], and development of severe depressive symptoms [6, 7]. A study on the emotional status of institutionalized older adults in Taiwan found 31.3–94.2% had depression [8], while the incidence of depression in older adults living in communities was only 8.8–15.3% [9]. Depression can lead to irritability, anxiety, and somatic symptoms. Severe depressive symptoms can lead to feelings of isolation, recurrent thoughts of death, and even suicide [10].

Depression in older adults can impact physical and mental health. A negative association between depression and heart rate variability (HRV) has been reported [11, 12]; decreased HRV reflects reduced parasympathetic activity and increased sympathetic activity. Low HRV can result in arrhythmia, increased cardiovascular disease, and sudden cardiac death [12]. Depression is often accompanied by impaired cognitive function, which is an important risk factor for dementia [13, 14]. A previous meta-analysis of longitudinal studies showed that people with depression had a higher incidence of all-cause dementia and mild cognitive impairment than those without depression [15]. This may indicate that depression promoted cognitive decline.

One approach to reducing depression is the use of story theory, a middle-range nursing theory developed by Smith and Liehr [16]. The “Attentively Embracing Story” applies story theory to allow health caregivers and care-receivers to address health challenges. The process consists of three concepts: intentional dialog, connecting with self-in-relation, and creating ease [16, 17]. When care-receivers describe their health stories, health caregivers sensitively pay attention, and attentively listen; care-receivers share past life experiences, thoughts, and feelings. Care-receivers reflect on their perceptions of present health challenges, and caregivers help them change their negative thoughts, generate new meanings of life experiences, connect the “self” in relationship with others and the outside world, and recognize living in the present moment is filled with hopes and dreams, in order to achieve the goal of healing [18–21]. One example is a 70-year old patient who continued to experience chest pain, despite having undergone cardiac surgery 6 months earlier. Over four sessions, a nurse guided the patient’s thinking about recovery from negative thoughts to positive thoughts. The nurse offered no treatment plan, however the patient’s pain diminished as a result of having an attentive listener [17]. In another study, Liehr and colleagues used stories of health to understand the experiences of 51 survivors of the World War II attacks on Hiroshima and Pearl Harbor [21]. Participants described their health, recollections of their experiences on the day of the bombings and then explained how the war contributed to their current state of health. This process helped the survivors reflect on past experiences of suffering, create new meanings of life, and transcend to a state of healing [21].

Several studies have used Smith and Liehr’s story inquiry method to develop effective intervention programs [19, 20, 22–24] and these can result in self-management behaviours that have positive health outcomes [20, 24]. Other studies have used the “Attentively Embracing Story” to design intervention programs that create trust relationships between caregivers and care-receivers through non-judgmental intentional dialogue, to help care-receivers discover unique life experiences [22, 23] or reduce stress [19].

Depression is high for older adults living in long-term care facilities [2, 8] and an intervention program for these older adults could decrease depressive symptoms. Therefore, we developed a story-centred care intervention program, based on the “Attentively Embracing Story” theory, for older adults living in an institutionalized setting. Our study aim was to evaluate the effectiveness of this form of intervention on reducing depressive symptoms, and improving cognitive function and HRV in older adults living in a long-term care facility.

## Materials and methods

### Study setting and participants

We recruited older adult residents of two long-term care facilities in Northern Taiwan through posters and notifications during routine activities and gatherings. Inclusion criteria for residents were: (1) older than 65 years; (2) with clear consciousness, normal hearing, and capable of conversation; (3) not taking any antidepressants at the time of the study; (4) without a clinical diagnosis of dementia or a score of 5 or greater on the Short Portable Status Questionnaire; (5) without loss of a loved one in the past three months; and (6) willing to participate in the study. Residents were excluded if they did not provide informed consent, or had cognitive impairment or medical illness that could interfere with treatment. The study was approved by the Institutional Review Board of the Tri-Service General Hospital National Defence Medical Centre (1-101-05-090IRB) and registered in the ClinicalTrials.gov (NCT02965937) in November 2016. The participants were fully informed of the research content, objectives, expected benefits, any potential risks, and the rights of participants for security, privacy and dignity. Written informed consent was obtained from all study participants. All procedures were performed in accordance with the relevant guidelines and regulations.

### Study design

The study was a parallel-design, single-blind, random assignment study enrolling 70 old persons. Outcome evaluator was blinded to treatment condition. After receiving informed consent, participants were randomly assigned to the experimental group (intervention, IG) or the control group (CG) using a computer-generated randomization scheme (SPSS software Version 22.0) by study staff. The random allocation sequence was in a uniform 1:1 allocation ratio. All participants were asked not to reveal their allocation status until the end of the study. Participants in the IG received the “Story-Centred Care Intervention Program” once a week for four weeks; the CG received a health consultation from researchers once a week for four weeks. Measures of Geriatric Depression Scale, Short Portable Mental Status Questionnaire, and the five-minute heart rate variability were obtained at pre-intervention (baseline), post-intervention, and 1- and 3-months follow-up. The study was conducted between January and August 2013. All participants had the right to withdraw from this study at any time without penalty or loss of benefits.

### Intervention

Development of the Story-Centred Care Intervention Program was based on the “Attentively Embracing Story” theory presented by Smith & Liehr [18]. Two experts in qualitative research and two geriatric social workers from the long-term care facility evaluated the intervention procedure and formulated the interview form. Before conducting this study, we tested the intervention with five older adults from a long-term care facility and obtained positive feedback. Participants in the IG received the Story-Centred Care Intervention Program for 60–90 minutes once a week for four weeks.

A trained researcher facilitated the six-step intervention. Step 1 guided participants in developing a story to describe challenges of their health problems, including past experiences and expectations about coping with these problems. During Step 2, participants revised the story to identify their most important and influential health challenges through existing literature. Step 3 determined key moments of the health challenges through understanding the participant’s feelings about these challenges. Step 4 involved accepting the story episodes, which were recorded and consolidated into a plot theme for the entire story. Step 5 guided the participant in describing approaches that could motivate him/her to resolve existing health challenges. Step 6 encouraged the participant to resolve the challenges by forming new meanings of life to improve their overall well-being. To protect participants’ privacy, the "Story-Centred Care Intervention Program" was implemented in a quiet and comfortable environment. During the process, researchers did not interfere, but asked questions to help clarify any part of the health story that was vague.

### Measures

#### Primary outcome

The primary outcome, change in severity of depressive symptoms over time, was assessed using the 15-Item Geriatric Depression Scale (GDS-15), at all assessment points (baseline, post-intervention and 1- and 3-month follow-up). We used the 15-Item Geriatric Depression Scale (GDS-15) to assess depressive symptoms. The GDS-15 is a self-administered questionnaire with a yes/no response. This study used the Chinese version of the GDS-15. A higher score indicates more severe depression symptoms; sensitivity is 70.6%, specificity is 70.1% for older adults [25].

#### Secondary outcome

The secondary outcomes, change in cognitive function and HRV over time, were assessed using the Short Portable Mental Status Questionnaire (SPMSQ) and the CheckMyHeart handheld HRV device at all assessment points (baseline, post-intervention and 1- and 3-month follow-up). The Short Portable Mental Status Questionnaire (SPMSQ) was used to evaluate cognitive function. The SPMSQ assesses disorientation, personal profile, short- and long-term memory, and computing ability. The number of incorrect answers indicates the level of cognitive function. This study used the Chinese version of the SPMSQ [26]. To fit the context of this study, some items were deleted, resulting in a 10-item questionnaire scored as follows: 0–2, intact cognitive function; 3–4, mild cognitive impairment; and ≥ 5, moderate to severe cognitive impairment. The test-retest reliability of the Chinese SPMSQ for older adults was 0.7 [26].The CheckMyHeart handheld HRV device (DailyCare BioMedical, Inc., Chungli, Taiwan) which is a limb-lead ECG (modified lead I) recorder with HRV analytical software and CE certified, has been used to measure HRV in previous studies [27, 28]. We used HRV time-domain parameters of standard deviation of the normal-to-normal intervals (SDNN), and root mean square of successive differences (RMSSD) as outcome variables, which are more strongly associated with psychological measures than frequency-domain [29]. Heart rate variability (HRV) refers to the situation whereby the heartbeat and heartbeat interval change. Most people's heart rate does not beat at a fixed speed. With careful measurement, it is found that each heartbeat and heartbeat intervals have small differences within a few dozen milliseconds. Even if people are in a calm and steady state, there is still a difference, and this difference is called the heart rate variability [30]. Heart rate variability is a simple and non-invasive method for assessing the function of the autonomic nervous system and can be classified into the time-domain analytical approach and the frequency-domain analytical approach. Time domain analysis is the statistical calculation of the variability of the heartbeat interval. This study used SDNN and RMSSD of time-domain analysis as the parameters of HRV. SDNN means the standard deviation of the normal-to-normal (NN) intervals, i. e. the square root of variance. SDNN reflects all cyclic components responsible for HRV in the period of recording. RMSSD, the square root of the mean squared differences of successive NN intervals, is regarded as the estimate for the short-term components of HRV. These measurements estimate high frequency variations in heart rate [30]. A depressed HRV can be regarded as a predictor to identify individuals at risk for subsequent morbid and mortal events. Demographic information was collected at baseline to characterize the sample. Socio-demographic data, health behaviors, and health status were collected with a Personal Information Form developed for this study. Participants’ satisfaction with social support was determined by a 14-item Chinese version of the Inventory of Social Supportive Behaviors scale [31]; a higher score indicates a higher degree of satisfaction with social support.

### Statistical analysis

The SPSS 22.0 software package, Chinese version, was used to analyse the data. We used descriptive statistics (mean, standard deviation, frequency, and percentage) to analyse participant characteristics and primary outcomes, and the Mann-Whitney U test to examine the initial differences between groups for demographics, depression status, cognitive function, and HRV measures. Generalized estimating equation (GEE) was used to examine the effects of the "Story-Centred Care Intervention Program" on improving depressive symptoms, cognitive function, and HRV. We recruited a total of 70 people and at the end analyzed the data of 60 people. Power was estimated using a repeated-measures MANOVA approach, under a within-between interaction in G*power 3.0.10 with type 1 error α = 0.05, effect size η^2^ = 0.43, and sample size = 60. The statistical power of this study was 0.78. Gay (1992) stated that when an experimental study is properly designed with rigorous experimental controls, each group requires at least 15 subjects [32]. In this study, we detected a statistically significant difference between the intervention group and the control group. Therefore, the sample size of 60 people in this study is acceptable. All tests were 2-tailed and α<0.05 was considered statistically significant.

## Results

### Participant characteristics

**Fig 1:**
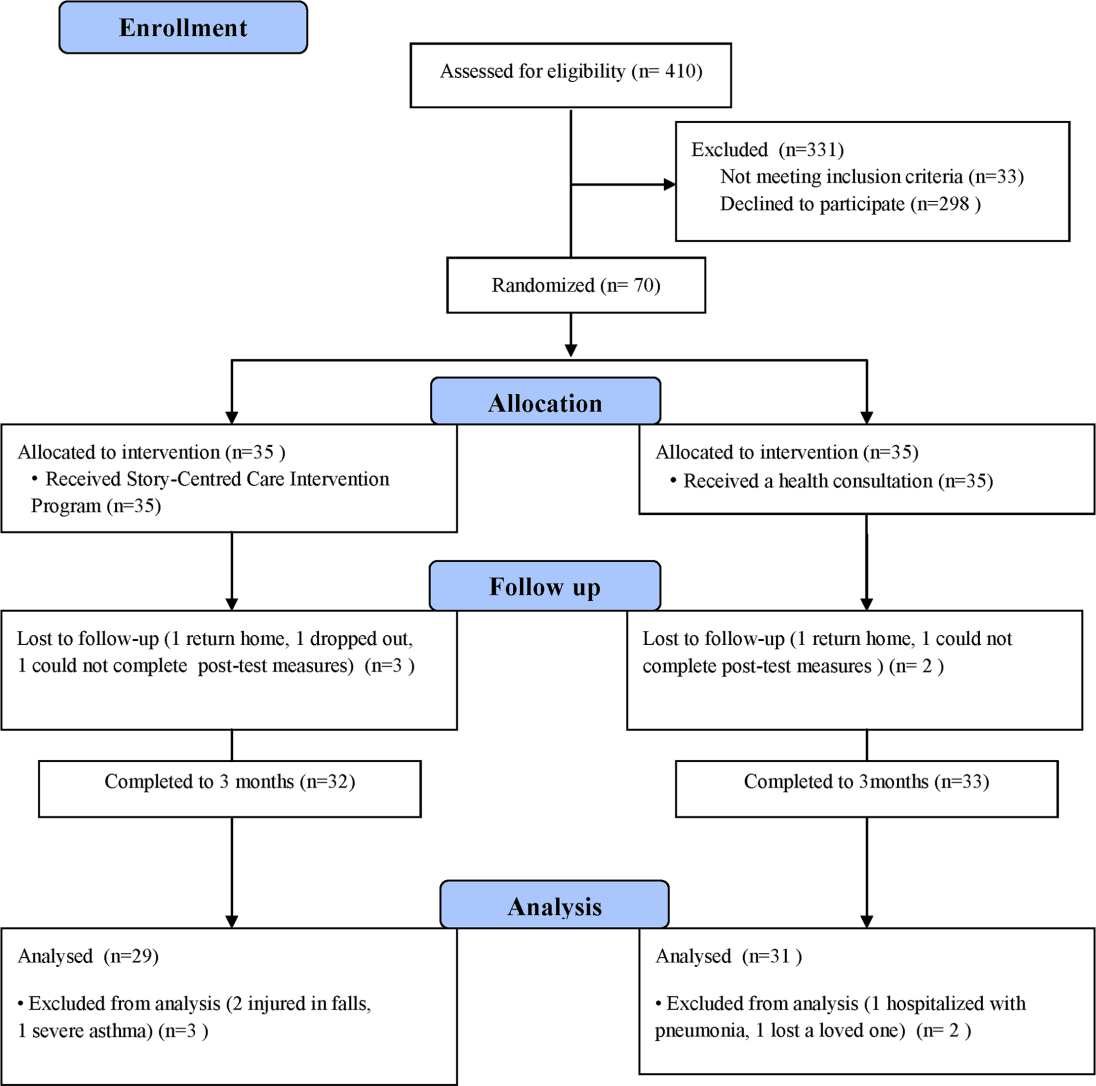
Consort flow diagram.

We recruited 70 long-term care residents for this study: two returned home, one dropped out, and two could not complete post-test measures due to surgery following a fall. Patients who completed treatment and those who dropped out were no differences in baseline demographic or clinical characteristics. Sixty-five participants completed the study. However, two were injured in falls, one was hospitalized with pneumonia, one visited the emergency room for severe asthma, and one lost a loved one; these were not included in the data analysis to avoid interference of events on outcome measures. Therefore, this study analysed data for 60 participants: 29 in the IG and 31 in the CG (Fig 1).

Participant characteristics are shown in Table 1; most participants were female (n = 40, 66.7%), with a mean age of 84.3±5.98 years. Most participants had a primary school degree (n = 20, 33.3%) and were widowed (n = 44, 73.3%).

**Table 1:** Baseline demographics, clinical characteristics, and health measures of participants.

Variable	Total(N = 60)	Participants (N = 60)		*X*^2^/Z	*P*
		IG (n = 29)	CG (n = 31)		
**Baseline demographics and clinical characteristics**					
Gender, n (%)				0.409^a^	0.523
Male	20 (33.3)	8 (27.6)	12 (38.7)		
Female	40 (66.7)	21 (72.4)	19 (61.3)		
Age, mean (SD)	84.30(5.98)	84.38 (4.74)	84.23 (7.03)	-0.200^b^	0.841
Education, n (%)				6.726^c^	0.081
Primary school	20 (33.3)	6 (20.7)	14 (45.2)		
Junior high school	9 (15)	7 (24.1)	2 (6.5)		
Senior high school	19 (31.7)	11 (38)	8 (25.8)		
≥ College	12 (20)	5 (17.2)	7 (22.6)		
Marital status, n (%)				1.359^c^	0.715
Single	4 (6.7)	2 (6.9)	2 (6.5)		
Married	12 (20)	6 (20.7)	6 (19.4)		
Widowed	44 (73.3)	21 (72.4)	23 (74.2)		
Smoking				2.954^d^	0.238
No	57 (95)	29 (100)	28 (90.3)		
Yes	3 (5)	0 (0.0)	3 (9.7)		
Drinking, n (%)					
No	60 (100)	29 (100)	31 (100)		
Yes	0	0 (0.0)	0 (0.0)		
Exercise, n (%)				0.285^d^	0.594
No	3 (5)	1 (3.4)	2 (6.5)		
Yes	57 (95)	28 (96.6)	29 (93.5)		
Physical function, n (%)				4.540^a^	0.033
Unassisted activity	43 (71.7)	25 (86.2)	18 (58.1)		
Assisted activity (using a device)	17 (28.3)	4 (13.8)	13 (41.9)		
Perceived health status, n (%)				1.696^c^	0.638
Very poor	9 (15)	4 (13.8)	5 (16.1)		
Poor	15 (25)	6 (20.7)	9 (29.0)		
Good	20 (33.3)	12 (41.4)	8 (25.9)		
Very good	16 (26.7)	7 (24.1)	9 (29.0)		
Social support satisfaction, mean (SD)	51.33 (7.84)	53.41(8.10)	49.39 (7.19)	-2.032^b^	0.042
**Health measures pre-intervention**					
GDS-15 score, mean (SD)	2.9 (3.21)	2.86 (2.94)	2.94 (3.50)	-0.150^b^	0.881
SPMSQ score, mean (SD)	0.58 (0.85)	0.66 (0.94)	0.52 (0.77)	-0.423^b^	0.673
SDNN, ms, mean (SD)	32.19 (28.32)	28.66 (19.72)	35.50 (34.50)	-0.377^b^	0.706
RMSSD, ms, mean (SD)	24.41 (25.01)	25.52 (22.21)	29.12 (27.63)	-0.599^b^	0.549

Abbreviations: IG, Intervention Group; CG, Control Group; SD, standard deviation; GDS-15, 15-item Geriatric Depression Scale; SPMSQ, short portable mental status questionnaire; SDNN, standard deviation of the normal-to-normal intervals; RMSSD, root mean square of successive differences.

^a^Yates continuity correction

^b^Mann-Whitney U test

^c^Pearsons’ chi-squared test

^d^Fisher’s exact test.

The IG and CG did not differ in gender, age, education, marital status, Smoking/drinking, exercise, perceived health status, and Health measures pre-intervention. However, significantly more participants in the IG than in the CG were unassisted activity (p = 0.033) and higher levels of social support satisfaction (p = 0.042). Most participants in the IG moved without assistance from a device (n = 25, 86.2%).The participants in IG perceived more satisfaction with social support than that of CG.

### Effect on reducing depressive symptoms

The GDS-15 was used to measure the effect of the intervention on depressive symptoms. After adjusting for baseline differences, the GEE model revealed a significant decrease in scores for the IG compared with the CG post-intervention (β = -1.612, χ^2^ = 13.257, p < .001; 95% CI = 0.08–0.48) and at 1 and 3 months follow-up (β = -1.621, χ^2^ = 11.951, p = .001, 95% CI = 0.08–0.50; β = -1.816, χ^2^ = 7.644, p = .006, 95% CI = 0.05–0.59, respectively) (Table 2). These findings suggested the intervention program improved depressive symptoms in older adults living in a long-term care facility.

**Table 2:** GEE analysis of the effect of the story-centered care intervention program on health outcome variables: depressive symptoms and cognitive function (N = 60).

Variable	Regression coefficient	Standard Error	X^2^	*p*-value	95% confidence interval
**GDS-15 score**					
Group (IG)^a^	0.464	0.805	0.332	0.564	0.33–7.70
Time (second)^b^	0.129	0.257	0.252	0.616	0.69–1.88
Time (third) ^b^	0	0.258	< 0.001	1.000	0.60–1.66
Time (fourth)^b^	0.161	0.498	0.105	0.746	0.44–3.12
Group (IG) x Time (second)^c^	-1.612	0.443	13.257	< .001	0.08–0.48
Group (IG) x Time (third)^c^	-1.621	0.469	11.951	0.001	0.08–0.50
Group (IG) x Time (fourth)^c^	-1.816	0.657	7.644	0.006	0.05–0.59
**SPMSQ**					
Group (IG)^a^	0.311	0.206	2.268	0.132	0.91–2.05
Time (second)^b^	-0.065	0.078	0.681	0.409	0.80–1.09
Time (third) ^b^	0.065	0.111	0.337	0.562	0.86–1.33
Time (fourth)^b^	0	0.091	<0.001	1	0.84–1.20
Group (IG) x Time (second)^c^	-0.073	0.101	0.528	0.467	0.76–1.13
Group (IG) x Time (third)^c^	-0.409	0.158	6.730	0.009	0.49–0.91
Group (IG) x Time (fourth)^c^	-0.345	0.153	5.114	0.024	0.53–0.96

Abbreviations: GEE, generalized estimating equation; IG, Intervention Group; 95% CI, 95% confidence interval; GDS-15, 15-item Geriatric Depression Scale; SPMSQ, short portable mental status questionnaire; first, pre-intervention; second, post-intervention; third, one month follow up; fourth, three months follow up.

^a^Reference group, Control group

^b^Reference group, Time (first)

^c^Reference group, Control group x Time (first).

### Effect on improvement of cognitive function

The SPMSQ evaluated improvement in cognitive function. After adjusting for baseline differences, the GEE model indicated that there was no significant difference in decrease in SPMSQ scores between the two groups post-intervention (β = -0.073, χ^2^ = 0.528, p = .467; 95% CI = 0.76–1.13). However, scores were significantly reduced for the IG compared to CG at 1 and 3 months follow-up (β = -0.409, χ^2^ = 6.73, p = .009, 95% CI = 0.49–0.91; β = -0.345, χ^2^ = 5.114, p = .024, 95% CI = 0.53–0.96, respectively) (Table 2). The findings revealed a significant improvement in cognitive function in the older adults who received the intervention program.

### Effect on improvement of HRV

**Fig 2:**
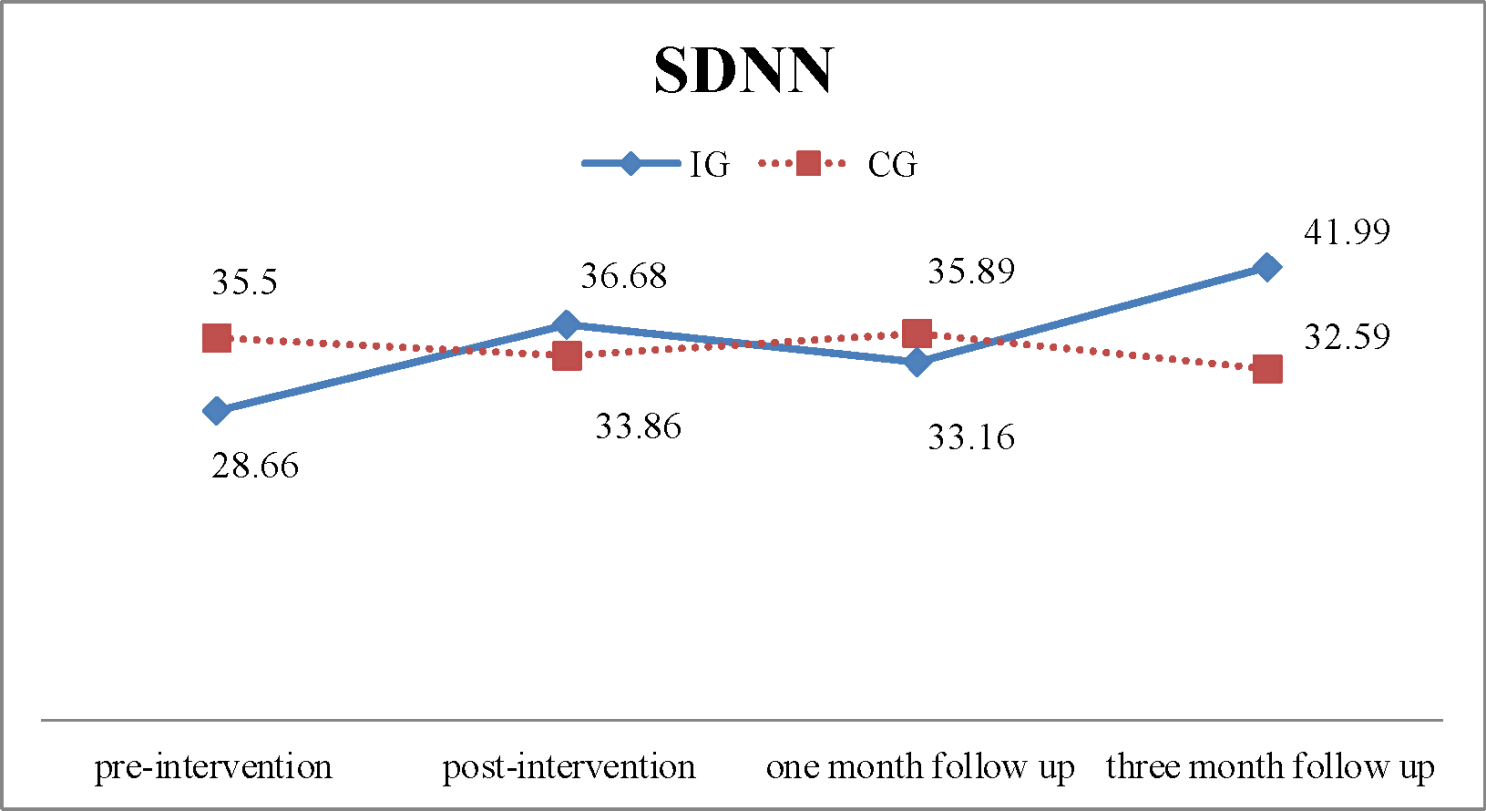
Time-domain parameters of heart rate variability for the intervention group (IG) and control group (CG): SDNN, standard deviation of the normal-to-normal intervals.

**Fig 3:**
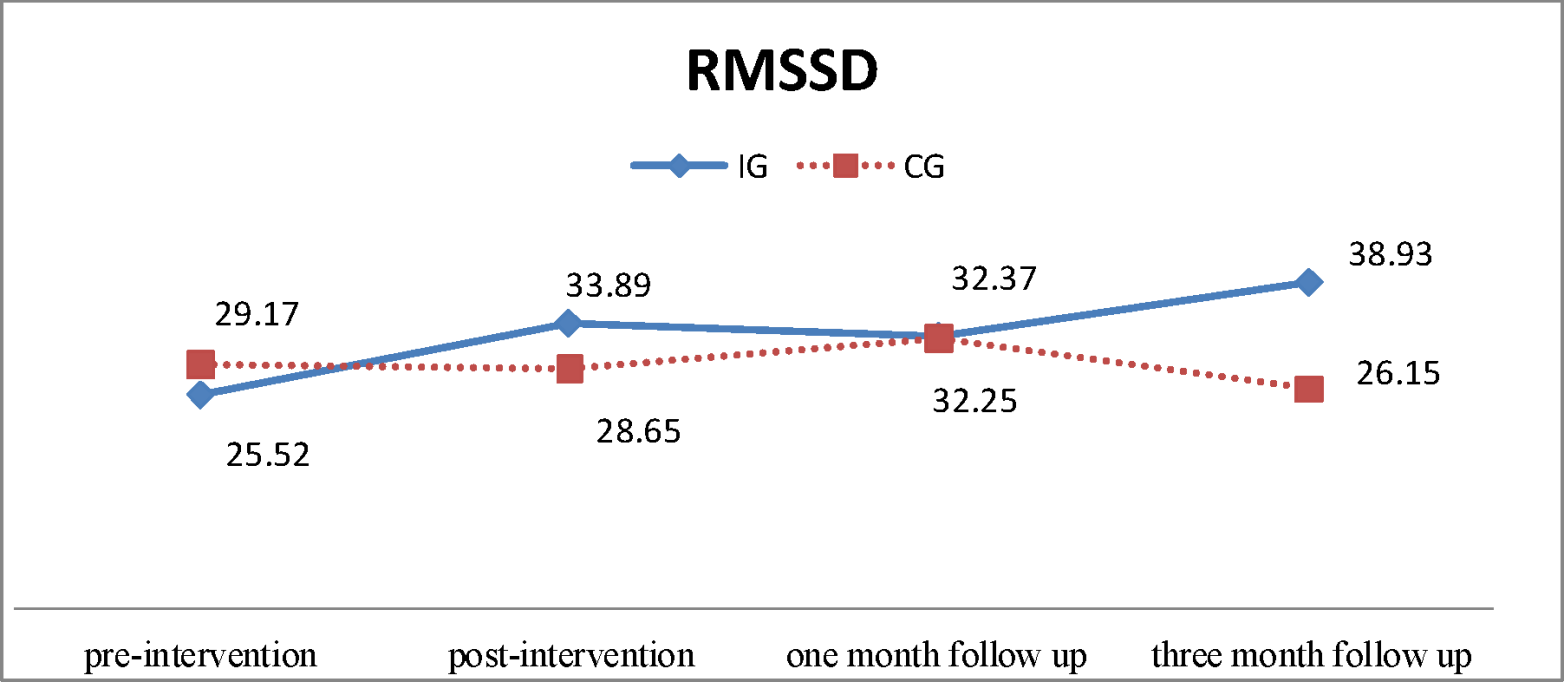
Time-domain parameters of heart rate variability for the intervention group (IG) and control group (CG): RMSSD, root mean square of successive differences.

After adjusting for baseline differences, the GEE model showed that SDNN did not differ significantly between groups post-intervention (β = 9.658, χ^2^ = 2.74, p = .098) or at 1 month and 3 months follow-up (β = 4.114, χ^2^ = 0.382, p = .536; β = 16.235, χ^2^ = 3.671, p = .055, respectively). However, there was a trend in an increase in SDNN in the IG, which was greater than that in the CG (Table 3, Figs 2 and 3). Again, after adjusting for baseline differences, the GEE model revealed that there was no significant difference in increase in RMSSD between groups post-intervention (β = 8.880, χ^2^ = 1.916, p = .166), or at 1 month and three months follow-up (β = 3.528, χ^2^ = 0.228, p = .633; β = 16.424, χ^2^ = 3.470, p = .062, respectively). However, similar to the SDNN, there was a trend in greater increase in RMSSD in the IG than the CG (Table 3, Figs 2 and 3).

**Table 3:** GEE analysis of the effect of the story-centered care intervention program on health outcome variables: heart rate variability (N = 60).

Variable	Regression coefficient	Standard Error	X^2^	*p*-value
**SDNN**				
Group (IG)^a^	-4.440	5.710	0.605	0.437
Time (second)^b^	-1.635	3.392	0.232	0.630
Time (third) ^b^	0.390	3.646	0.011	0.915
Time (fourth)^b^	-2.908	3.737	0.606	0.436
Group (IG) x Time (second)^c^	9.658	5.834	2.740	0.098
Group (IG) x Time (third)^c^	4.114	6.654	0.382	0.536
Group (IG) x Time (fourth)^c^	16.235	8.474	3.671	0.055
**RMSSD**				
Group (IG)^a^	-3.398	5.393	0.397	0.529
Time (second)^b^	-0.517	3.302	0.025	0.875
Time (third) ^b^	3.203	3.881	0.681	0.409
Time (fourth)^b^	-3.020	3.620	0.696	0.404
Group (IG) x Time (second)^c^	8.880	6.416	1.916	0.166
Group (IG) x Time (third)^c^	3.528	7.389	0.228	0.633
Group (IG) x Time (fourth)^c^	16.424	8.817	3.470	0.062

Abbreviations: GEE, generalized estimating equation; IG, Intervention Group; SDNN, standard deviation of the normal-to-normal intervals; RMSSD, root mean square of successive differences; first, pre-intervention; second, post-intervention; third, one month follow up; fourth, three months follow up.

^a^Reference group, Control group

^b^Reference goup, Time (first)

^c^Reference group, Control group x Time (first).

## Discussion

This study developed an intervention program based on the “Attentively Embracing Story” theory to examine the effects of a “Story-Centred Care Intervention Program” on improving depressive symptoms, cognitive function, and HRV in older adults living in a long-term care facility. Four weeks after the intervention, participants in the IG showed a significant decrease in depression, improvement in cognitive function, and a trend towards improved SDNN and RMSSD compared with the CG.

Participants who received the intervention program showed a significant decrease in GDS-15 scores at all three time points compared with controls. The results indicate the "Story-Centred Care Intervention Program" reduced depression and the effects were sustained for up to 3 months. Our findings are similar to Crogan et al. [19] who showed a nurse-led storytelling intervention improved depression in cancer patients; participants discovered a new meaning for life through storytelling in spite of a terminal disease. Our participants had similar experiences and reactions. Presentation of their stories resulted in strengthened self-awareness, reduced emotional distress, and fewer depressive symptoms. Based on our results, the story-centred care intervention program could reduce depression in older adults living in long-term care facilities.

Greater decreases in SPMSQ scores for the IG compared with the CG revealed the intervention improved older adults’ cognitive function. Participants remembered past experiences, thoughts, and feelings in a positive self-reflective manner, which enabled them to accept life challenges in the present moment. The findings are similar to the process of reminiscence and life review [33]. Recollection of relevant past events restores forward memories, stimulates long-term and short-term memory functions, and improves orientation [28, 33]. Our findings suggest that implementation of the intervention program could reduce cognitive impairment and improve cognitive function in older adults living in long-term care facilities.

There was a trend towards improved HRV measures of SDNN and RMSSD for participants in the IG, although group differences were not statistically significant. HRV in older adults is associated with depression [34] and a recent study showed a negative correlation between depression and HRV in post-cardiac surgery patients; persons with depression had significantly lower SDNN and RMSSD than those without depression [35]. Although the intervention reduced depression, the lack of significant differences in HRV measures could be a result of the mild depressive status at baseline; the GDS-15 score was < 5 for both groups. Psychological factors such as depression are negatively associated with autonomic function only when they reach a critical threshold [29]. In addition, the four-month study period might also contribute to the small change in HRV. Although there was no significant improvement in HRV following the intervention program, depression was reduced and there was a trend in increased HRV measures.

## Limitations

This study had limitations. Recruiting in areas where routine daily activities occurred might select for healthier residents, which may have excluded persons with poor physical function or greater depression. Second, most of the outcomes were measured with self-reported questionnaires. Third, participants were recruited from two long-term care facilities in northern Taiwan, and the results may not be generalizable to a larger population.

## Conclusion

The “Attentively Embracing Story” theory has been tested primarily in qualitative studies. We developed a “Story-Centred Care Intervention Program” based on the theory and used quantitative measures to examine improvements in depression, cognitive function and HRV. This program could be adopted as an intervention with older adults in outpatient care to address their healthcare concerns, reduce depression and improve cognitive function. Whether this intervention can improve HRV will require additional studies. Some recommendations are as follows. First, the measurement of this study mostly adopted the subjective questionnaire survey method. In the future, researchers can use more objective values, so as to make the results more objective and to increase the credibility of intervention effectiveness. Second, this study only recruited elderly people from two long-term care institutions in northern Taiwan as the subjects. It is not clear to us whether there the same effect arises when the story-centred care intervention program is implemented in different institutions. It is suggested that future studies can conduct similar small-scale trials in different institutions, or expand the sample size to carry out larger trials through the cooperation of multiple institutions, so as to enhance the inference of external validity.

## Supporting information

S1 Fig Study design. (TIF)

S1 Table Outcome measurements. (TIF)

S1 File Consort checklist. (DOC)

S2 File Trial protocol. (DOC)

S3 File Raw data. (XLS)
