# Supplementary figures and images for: Effectiveness of Story-Centred Care Intervention Program in older persons living in long-term care facilities: A randomized, longitudinal study
Source: PLoS One. 2018 Mar 19;13(3):e0194178. doi: 10.1371/journal.pone.0194178 (PMC5858786; doi:10.1371/journal.pone.0194178)

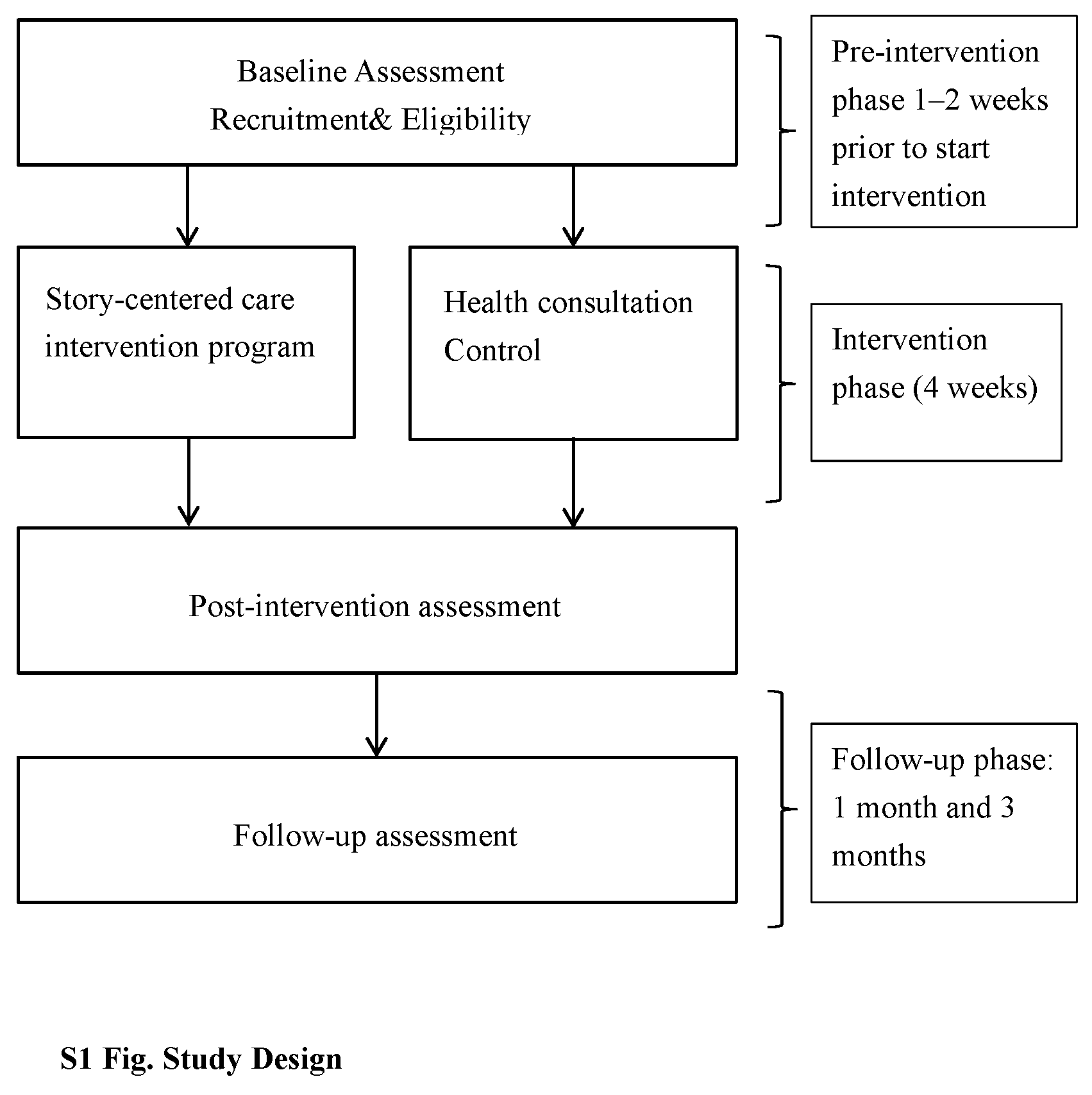

Supplement: S1 Fig — (TIF) [file pone.0194178.s001.tif]

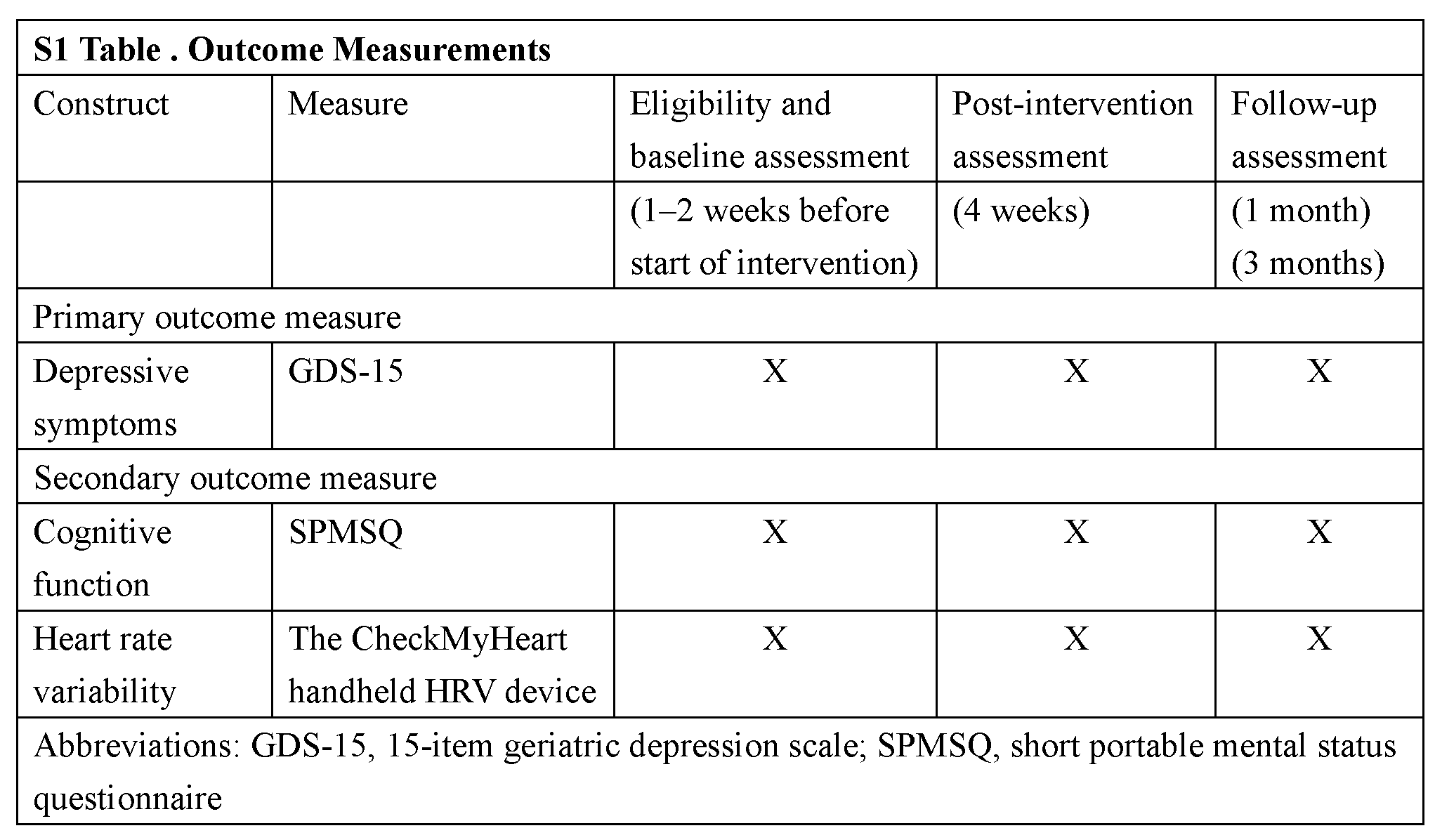

Supplement: S1 Table — (TIF) [file pone.0194178.s002.tif]
