# Supplementary material for: Effectiveness of Story-Centred Care Intervention Program in older persons living in long-term care facilities: A randomized, longitudinal study
Source: PLoS One. 2018 Mar 19;13(3):e0194178. doi: 10.1371/journal.pone.0194178 (PMC5858786; doi:10.1371/journal.pone.0194178)
Supplement: S2 File — (DOC) [file pone.0194178.s004.doc]

**Supplement : Trial Protocol**

**A. Background**

Depression is a common psychological problem in older adults living in long-term care facilities^1^. Depression is associated with high rates of chronicity, poor quality of life and impairments in physical, cognitive and social functioning as well as high risk of morbidity and mortality in the elderly with chronic medical disorders^2^.

Story-centred care intervention is a theory-guided approach that emphasises a health-promoting potential of nurse–person dialogue about a health challenge. The assumptions made by the theory of attentively embracing story are that people (1) change in multidimensional arrays as they interrelate with their world, (2) live in an expanded present moment that comprises past and future events and (3) experience meaning regarded as a form of an unfolding of their potential^3^. When care receivers (storytellers) describe their complex health stories, health caregivers (story gatherers) sensitively pay attention and attentively listen to the storytellers’ narration about their past life experiences, thoughts and feelings^3^. During this time, the story gatherers allow the storytellers to reflect on their perceptions of present health challenges. In addition, story gatherers help storytellers in changing their negative thoughts on events, generating new meanings from life experiences, connecting the ‘self’ in relationships with others and the outside world and recognising that he/she is living in the present moment filled with hope and dreams; consequently, achieve their goal of healing^3^.

Story-centred care intervention, based on the theory of attentively embracing story, is composed of three concepts. First, intentional dialogue, which is querying emergence in the true presence, is defined as purposeful engagements with others. Intentional dialogue gathers a story of complicating health challenges in the true presence. The story gatherers provide non-judgmental, rhythmical focusing as well as a refocusing of energy to the storytellers and pose questions to clarify meaning^3^. Second, connecting with self-in-relation, which is reflective awareness of personal history, is defined as an active process where the storyteller recognises herself or himself as related with others in a developing story^3^. It means that one’s personal history integrates with a reflective awareness concerning the story. When one tells a story about his or her own health experience and reflects on that experience, storytellers and story gatherers may generate a new meaning from the health experience. The third concept of creating ease is remembering disjointed story moments with a flow of experience in the midst of anchoring, defined by Smith and Liehr (1999)^4^ as ‘an energising release experienced as the story comes together in a movement toward resolving’. This concept emphasises that when the storytellers find new meaning from their personal stories of health challenges, a renewed sense of purpose is achieved, resulting in ease and facilitating spiritual healing.

According to previous studies, story-centred care intervention has some benefits. First, it can help participants to confirm challenges posed by their health problems and motivate them to resolve existing health challenges^5,6^. Second, it can create trusting relationships between story gatherers and storytellers, which can transform storytellers’ sufferings and help them find meaning in their lives^7-10^. Third, it can result in self-management behaviours that have positive health outcomes^5,11^.

Despite the effectiveness of story-centred care intervention, most of the previous studies were qualitative in nature^5,6,8-10^. Few studies were quantitative but their sample sizes were small^7,11^. Therefore, we intend to conduct a parallel-design, random assignment study involving enrolment of 60 patients and collecting a large number of data points for each participant at each measurement, with four repeated measurements over a 3-month follow-up period.

**B. Objective**

The aim of this study is to evaluate the effectiveness of a story-centred care intervention program on reducing depressive symptoms and improving cognitive function and heart rate variability (HRV) for older adults living in a long-term care facility.

**C. Methods**

**Design**

A single-blind two-group longitudinal experimental study will be conducted to compare a story-centred care intervention program with a health consultation control condition. After receiving informed consents, participants will be randomly assigned to one of the two treatment conditions, either a story-centred care intervention program or a health consultation control condition. Each intervention will be conducted for 4 weeks. Measurements will be obtained at the following time points: pre-intervention (baseline), post-intervention and 1-month- and 3-month follow-up (see S1 Fig.). Outcome measurements include the following parameters: 15-item geriatric depression scale test (GDS-15), short portable mental status questionnaire (SPMSQ) and 5-minute HRV.

**Inclusion and exclusion criteria**

We will include older adults based on the following criteria: (1) those who are ≥65 years old and live in long-term care facilities, (2) have clear consciousness and normal hearing and conversation capability, (3) are not taking any antidepressants at the time of the study, (4) have not lost a loved one in the past 3 months and (5) are willing to participate in the study. We will exclude participants who (1) meet DSM-IV criteria for Dementia of the Alzheimer’s type, (2) suffer from moderate to severe cognitive impairment (SPMSQ > 5), (3) are taking antidepressants at the time of the study, (4) are undergoing other psychotherapy during the study and (5) have any underlying medical illness that could interfere with the treatment.

**Recruitment**

Participants will be recruited from January 2013 to August 2013 via posters and notifications during routine activities and gatherings. The study design and protocols will be reviewed and approved by the institutional review board of the participating hospital. At the content session, all assessments and procedures will be fully explained. When participants express their interest in participating in the study, they need to leave their contact information at the front desk. The research team will contact the participants as soon as a list is compiled.

**Assessment of eligibility and randomisation**

Participants will beconsidered eligible if they meet the inclusion criteria. All participants will be informed that they can withdraw themselves from the study at any time. Before randomisation, participants will be asked to answer a few questionnaires and will be subjected to the 5-minute HRV test. The questionnaires include demographic characteristics, the 15-item geriatric depression scale test (GDS-15) and SPMSQ. Randomisation will be conducted using a computer-generated randomisation scheme (SPSS software Version 22.0). Participants will be randomly assigned to the story-centred care intervention program or to the health consultation control condition. The random allocation sequence will be set in a uniform 1:1 allocation ratio.

**Blinding**

Participants will be randomly assigned to either the story-centred care intervention program or the health consultation control condition. They will be informed not to disclose their randomisation status to anyone. The outcome evaluator will be blinded to the assigned condition of the participants.

**Intervention**

Participants will randomly be assigned to the experimental (intervention) group (IG) or the control group (CG) using SPSS software (Version 22.0). Participants in the IG will receive the story-centred care intervention program once a week for 4 weeks; the CG will receive health consultation from researchers once a week for 4 weeks.

***Story-centred care intervention program***

Participants randomised to the IG will be made to participate in a 4-week long story-centred care intervention program conducted by a trained researcher. A trained researcher will facilitate the six-step intervention. Step 1 involves gathering stories about complicated health challenges of the participants. The researcher will guide the participants in describing their health challenges using elements of a story. The challenges include current health problems, past experiences related to these problems and expectations for dealing with these problems. During Step 2, connections of the health challenges to existing literature will be made and a story is reconstructed. In order to ensure that the health challenge is a true reflection of participants’ condition, the researcher identifies the complicated health challenges of the participants through existing literature and helps them revise the story to identify their most important and influential health challenges. Step 3 determines key moments of the health challenges by understanding the participants’ feelings about these challenges. Step 4 involves accepting the story episodes, which will be recorded and consolidated into a theme for the entire story. In Step 5, the researcher guides the participant to describe approaches that can motivate him/her to resolve existing health challenges. In Step 6, the participant is encouraged to resolve the challenges by generating new meanings of life to improve their overall well-being. During the process, the researcher does not interfere but asks questions to help the participants clarify vague parts of the health story.

***Health consultation control condition***

Participants randomised to the control group will receive a health consultation from a trained researcher once a week for 4 weeks. The health consultation will be tailored to the participants’ needs. Based on a previous study^12^, the health consultation will include pain management, healthy nutrition, how to make best use of medical services and education and counselling for individual health-related concerns.

**Statistical analysis**

We will use descriptive statistics (mean, standard deviation, frequency and percentage) to analyse participant characteristics and primary outcomes. Mann–Whitney U test is used to examine the continuous variables between groups and chi-square test (or Fisher’s Exact tests where appropriate) is used to examine the categorical variables between groups. Generalised estimating equation (GEE) is used to examine the effects of the story-centred care intervention program on the improvement of depression symptoms, cognitive function and HRV. The SPSS 22.0 software package, Chinese version, will be used to analyse the data. All tests are two-tailed and P values < 0.05 is considered statistically significant.

**Outcomes measurements**

For an overview of assessment at baseline, post-intervention and 1- and 3-month follow-up, refer to S1 Table.

***Primary outcome***

**15-item geriatric depression scale test (GDS-15):** The GDS-15 test assesses depressive symptoms. The GDS-15 test is a self-administered questionnaire with a yes/no response. This study used the Chinese version of the GDS-15 test. A higher score indicates more severe depression with a sensitivity of 70.6% and a specificity of 70.1% for older adults^13^.

***Secondary outcome***

**The short portable mental status questionnaire (SPMSQ):** The SPMSQ assesses disorientation, personal profile, short- and long-term memory and computing ability. The number of incorrect answers indicates the level of cognitive function. This study uses the Chinese version of the SPMSQ^14^. To suit the context of this study, some items are deleted, resulting in a 10-item questionnaire scored as follows: 0–2, intact cognitive function; 3–4, mild cognitive impairment and ≥5, moderate to severe cognitive impairment. The test-retest reliability of the Chinese SPMSQ for older adults is 0.7^14^.

**Heart rate variability (HRV):** HRV is measured using the CheckMyHeart handheld HRV device (DailyCare BioMedical Inc., Chungli, Taiwan), which is a limb-lead ECG (modified lead I) recorder with HRV analytical software. It is CE certified in several countries (US, Canada, Japan and Taiwan) and has been used in previous studies^15,16^. We make use of HRV time-domain parameters of standard deviation of the normal-to-normal intervals (SDNN) and root mean square of successive differences (RMSSD) as outcome variables, which are more strongly associated with psychological measures than frequency domain^17^.

**References**

1 Dow, B., Lin, X., Tinney, J., Haralambous, B. & Ames, D. Depression in older people living in residential homes. *International Psychogeriatrics / IPA* **23**, 681-699, doi:10.1017/S1041610211000494 (2011).

2 Blazer, D. G. Depression in late life: review and commentary. *The Journals Of Gerontology. Series A, Biological Sciences And Medical Sciences* **58**, 249-265 (2003).

3 Smith, M. J. & Liehr, P. Story theory: advancing nursing practice scholarship. *Holistic Nursing Practice* **19**, 272-276 (2005).

4 Smith, M. J. & Liehr, P. Attentively Embracing Story: a middle-range theory with practice and research implications. *Scholarly inquiry for nursing practice* **13**, 187-204; discussion 205-110 (1999).

5 Hain, D. J., Wands, L. & Liehr, P. Approaches to resolve health challenges in a population of older adults undergoing hemodialysis. *Research in gerontological nursing* **4**, 53-62, doi:10.3928/19404921-20100330-01 (2011).

6 Jolly, K., Weiss, J. A. & Liehr, P. Understanding adolescent voice as a guide for nursing practice and research. *Issues in comprehensive pediatric nursing* **30**, 3-13, doi:10.1080/01460860701366518 (2007).

7 Crogan, N. L., Evans, B. C. & Bendel, R. Storytelling intervention for patients with cancer: part 2--pilot testing. *Oncology nursing forum* **35**, 265-272, doi:10.1188/08.onf.265-272 (2008).

8 Carlick, A. & Biley, F. C. Thoughts on the therapeutic use of narrative in the promotion of coping in cancer care. *European journal of cancer care* **13**, 308-317, doi:10.1111/j.1365-2354.2004.00466.x (2004).

9 Theeke, L. A. & Mallow, J. A. The Development of LISTEN: A Novel Intervention for Loneliness. *Open journal of nursing* **5**, 136-143, doi:10.4236/ojn.2015.52016 (2015).

10 Takahashi, R., Liehr, P., Nishimura, C., Ito, M. & Summers, L. C. Meaning of health for Japanese elders who have had a stroke. *Japan Journal of Nursing Science* **2**, 41-49, doi:10.1111/j.1742-7924.2005.00032.x (2005).

11 Liehr, P. *et al.* Adding story-centered care to standard lifestyle intervention for people with Stage 1 hypertension. *Applied Nursing Research* **19**, 16-21, doi:10.1016/j.apnr.2004.12.001 (2006).

12 Imhof, L., Naef, R., Wallhagen, M. I., Schwarz, J. & Mahrer-Imhof, R. Effects of an Advanced Practice Nurse In-Home Health Consultation Program for Community-Dwelling Persons Aged 80 and Older. *Journal of the American Geriatrics Society* **60**, 2223-2231, doi:10.1111/jgs.12026 (2012).

13 Chan, A. C. Clinical validation of the Geriatric Depression Scale (GDS) Chinese version. *Journal of Aging & Health* **8**, 238-253 (1996).

14 Chi, I. & Boey, K. Hong Kong validation of measuring instruments of mental health status of the elderly. *Clinical Gerontologist* **13**, 35-51 (1993).

15 Chen, J.-H., Chao, Y.-H., Lu, S.-F., Shiung, T.-F. & Chao, Y.-F. The effectiveness of valerian acupressure on the sleep of ICU patients: A randomized clinical trial. *International Journal of Nursing Studies* **49**, 913-920, doi:10.1016/j.ijnurstu.2012.02.012 (2012).

16 Ieda, M. *et al.* Evaluation of autonomic nervous system by salivary alpha-amylase level and heart rate variability in patients with schizophrenia. *European Archives Of Psychiatry And Clinical Neuroscience* **264**, 83-87, doi:10.1007/s00406-013-0411-6 (2014).

17 Francis, J. L. *et al.* Association between symptoms of depression and anxiety with heart rate variability in patients with implantable cardioverter defibrillators. *Psychosomatic Medicine* **71**, 821-827, doi:10.1097/PSY.0b013e3181b39aa1 (2009).

Baseline Assessment

Recruitment& Eligibility

Eligibility and baseline assessment

Story-centered care intervention program

Health consultation

Control

Post-intervention assessment

Follow-up assessment

Pre-intervention phase 1–2 weeks prior to start intervention

Intervention phase (4 weeks)

Follow-up phase:

1 month and 3 months

**S1 Fig. Study Design**

| **S1 Table. Outcome Measurements** | | | | |
| --- | --- | --- | --- | --- |
| Construct | Measure | Eligibility and baseline assessment | Post-intervention  assessment | Follow-up assessment |
|  |  | (1–2 weeks before start of intervention) | (4 weeks) | (1 month)  (3 months) |
| Primary outcome measure | | | | |
| Depressive symptoms | GDS-15 | X | X | X |
| Secondary outcome measure | | | | |
| Cognitive function | SPMSQ | X | X | X |
| Heart rate variability | The CheckMyHeart handheld HRV device | X | X | X |
| Abbreviations: GDS-15, 15-item geriatric depression scale; SPMSQ, short portable mental status questionnaire | | | | |
